# Ipilimumab plus nivolumab and chemoradiotherapy followed by surgery in patients with resectable and borderline resectable T3-4N0–1 non-small cell lung cancer: the INCREASE trial

**DOI:** 10.1186/s12885-020-07263-9

**Published:** 2020-08-14

**Authors:** Chris Dickhoff, Suresh Senan, Famke L. Schneiders, Joris Veltman, Sayed Hashemi, Johannes M. A. Daniels, Marieke Fransen, David J. Heineman, Teodora Radonic, Peter M. van de Ven, Imke H. Bartelink, Lilian J. Meijboom, Juan J. Garcia-Vallejo, Daniela E. Oprea-Lager, Tanja D. de Gruijl, Idris Bahce

**Affiliations:** 1 Department of Surgery and Cardiothoracic Surgery, Amsterdam University Medical Center, location VUmc, Cancer Center Amsterdam, de Boelelaan 1117, 1081HV Amsterdam, the Netherlands; 2 Department of Radiation Oncology, Amsterdam University Medical Center, location VUmc, Cancer Center Amsterdam, de Boelelaan 1117, 1081HV Amsterdam, the Netherlands; 3 Department of Pulmonary Diseases, Amsterdam University Medical Center, location VUmcCancer Center Amsterdam, de Boelelaan 1117, 1081HV Amsterdam, the Netherlands; 4 Department of Pathology, Amsterdam University Medical Center, location VUmc, Cancer Center Amsterdam, de Boelelaan 1117, 1081HV Amsterdam, the Netherlands; 5 Department of Epidemiology and Biostatistics, Amsterdam University Medical Center, location VUmc, Cancer Center Amsterdam, de Boelelaan 1117, 1081HV Amsterdam, the Netherlands; 6 Department of Clinical Pharmacology and Pharmacy, Amsterdam University Medical Center, location VUmc, Cancer Center Amsterdam, de Boelelaan 1117, 1081HV Amsterdam, the Netherlands; 7 Department of Radiology and Nuclear Medicine, Amsterdam University Medical Center, location VUmc, Cancer Center Amsterdam, de Boelelaan 1117, 1081HV Amsterdam, the Netherlands; 8 Department of Molecular Cell Biology & Immunology, Amsterdam University Medical Center, location VUmc, Cancer Center Amsterdam, de Boelelaan 1117, 1081HV Amsterdam, the Netherlands; 9 Department of Medical Oncology, Amsterdam University Medical Center, location VUmc, Cancer Center Amsterdam, de Boelelaan 1117, 1081HV Amsterdam, the Netherlands

**Keywords:** NSCLC, Neoadjuvant immunotherapy, Thoracic surgery, Locally advanced, Pathological response

## Abstract

**Background:**

The likelihood of a tumor recurrence in patients with T3-4N0–1 non-small cell lung cancer following multimodality treatment remains substantial, mainly due distant metastases. As pathological complete responses (pCR) in resected specimens are seen in only a minority (28–38%) of patients following chemoradiotherapy, we designed the INCREASE trial (EudraCT-Number: 2019–003454-83; Netherlands Trial Register number: NL8435) to assess if pCR rates could be further improved by adding short course immunotherapy to induction chemoradiotherapy. Translational studies will correlate changes in loco-regional and systemic immune status with patterns of recurrence.

**Methods/design:**

This single-arm, prospective phase II trial will enroll 29 patients with either resectable, or borderline resectable, T3-4N0–1 NSCLC. The protocol was approved by the institutional ethics committee. Study enrollment commenced in February 2020.

On day 1 of guideline-recommended concurrent chemoradiotherapy (CRT), ipilimumab (IPI, 1 mg/kg IV) and nivolumab (NIVO, 360 mg flat dose IV) will be administered, followed by nivolumab (360 mg flat dose IV) after 3 weeks. Radiotherapy consists of once-daily doses of 2 Gy to a total of 50 Gy, and chemotherapy will consist of a platinum-doublet. An anatomical pulmonary resection is planned 6 weeks after the last day of radiotherapy. The primary study objective is to establish the safety of adding IPI/NIVO to pre-operative CRT, and its impact on pathological tumor response. Secondary objectives are to assess the impact of adding IPI/NIVO to CRT on disease free and overall survival. Exploratory objectives are to characterize tumor inflammation and the immune contexture in the tumor and tumor-draining lymph nodes (TDLN), and to explore the effects of IPI/NIVO and CRT and surgery on distribution and phenotype of peripheral blood immune subsets.

**Discussion:**

The INCREASE trial will evaluate the safety and local efficacy of a combination of 4 modalities in patients with resectable, T3-4N0–1 NSCLC. Translational research will investigate the mechanisms of action and drug related adverse events.

**Trial registration:**

Netherlands Trial Registration (NTR): NL8435, Registered 03 March 2020.

## Background

Lung cancer remains a leading cause of cancer-related deaths, even though considerable progress has been made recently in systemic therapies for this entity [1, 2]. In patients presenting with metastatic non-small cell lung cancer (NSCLC), immunotherapy is now established as a standard of care in first line therapy, either alone or in combination with chemotherapy [3, 4]. In patients presenting with unresectable, non-metastatic locally advanced NSCLC, the administration of durvalumab (anti-PD-L1 antibody) consolidation for 12 months after definitive chemo-radiotherapy (CRT) has been shown to improve both overall and progression-free survival [5]. To further improve clinical outcomes, concurrent administration of immunotherapy with CRT in stage III NSCLC has been evaluated, and shown to be feasible for nivolumab and atezolizumab in the NICOLAS and DETERRED trials, respectively [6, 7]. The results of a completed phase 3 study assessing the efficacy and safety of durvalumab given concurrently with CRT in patients with locally advanced, unresectable NSCLC, are awaited [NCT03519971].

Multimodal strategies are recommended in the ESMO guidelines for fit patients with potentially resectable locally-advanced NSCLC [8]. With this strategy, both the local tumor and regional lymph node metastases are targeted using chemo-radiotherapy and surgery, and any occult distant metastases by chemotherapy. Surgery is preferred as a component of multimodality treatment in fit patients with large volume lung tumors, or tumors invading adjacent structures such as the chest wall and mediastinum. Although clinical outcomes have improved with the implementation of multi-multimodal strategies [9, 10], rates of distant metastases are still substantial, and pathological complete responses are seen in only a minority (28–38%) of patients [10–13]. The trimodality approach has been reported to be safe in terms of toxicity, morbidity and mortality, 5-year progression-free survival rates range from 22 to 32% [11, 12, 14]. Strategies to improve pathological complete response rates in this population may be useful as patients with < 10% of vital tumor after neoadjuvant therapy have been found to have an improved overall survival [10–13].

A growing body of evidence suggests that neoadjuvant administration of immune checkpoint inhibitors can achieve anti-tumor immune priming and/or boosting [15, 16]. In the presence of the pre-therapy tumor mass, a broader and stronger T-cell response can be induced, as suggested by preclinical and early clinical studies. In addition, the presence of activated tumor-specific T cells may prevent metastatic spread [15, 17].

### Chemotherapy and immunotherapy induction

The efficacy of induction therapy in NSCLC appears to increase with by adding immunotherapy. The NEOSTAR trial (NCT03158129), a phase 2 randomized study in stage I-IIIA NSCLC, combined neo-adjuvant ipilimumab (anti-CTLA-4) with nivolumab versus nivolumab alone, and both arms underwent resection with reported pathological complete response (pCR) rates of 44 and 19% in the ipilimumab-nivolumab (IPI/NIVO) and nivolumab arms, respectively [18]. The NADIM trial (NCT03081689), a phase 2 single arm study of resectable stage IIIA-N2 NSCLC treated with induction chemotherapy and nivolumab, followed by resection and adjuvant nivolumab for 1 year, also showed increased efficacy [19]. The authors reported a pCR rate of 71.4% (95%CI 54–87%), which represents a major improvement when compared to historic pCR rates of 5% with chemotherapy alone [9] and up to 40% with chemotherapy combined with radiotherapy [11, 12].

### Radiotherapy and immunotherapy induction

Combination treatment of neo-adjuvant (chemo) radiotherapy and immunotherapy might potentiate a synergistic effect. In addition to inducing irreparable DNA damage, ionizing radiation can trigger tumor cells to undergo immunogenic cell death, which in turn induces a pro-inflammatory immune response [20]. The advantage of neo-adjuvant treatment over adjuvant treatment lies in the fact that tumor cells are abundantly present at time of treatment, enabling the process of ‘in-situ vaccination’, in which dying tumor cells are engulfed by antigen presenting cells which in turn can drive tumor-specific memory T-cell responses [21]. This process has the potential to establish protection in case of microscopic disease or remaining solitary tumor cells after treatment, and diminishing chances of local residual disease as well as development of distant metastases. A phase II trial in NSCLC patients who were unresponsive to ipilimumab (anti–CTLA-4) alone, and who were treated with ipilimumab and RT, revealed an increased immunological response after combination treatment [22]. The addition of ipilimumab to radiotherapy was associated with an increase in abscopal effects, suggestive for activation of the immune system that led to responses in non-irradiated lesions.

### Toxicity of induction schemes using chemoradiotherapy and immunotherapy

Immunotherapy has been safely combined with platinum based chemotherapy and radiotherapy in clinical trials investigating locally advanced stage NSCLC [19, 23]. Neoadjuvant administration of immunotherapy may increase the risk of immune related Adverse Events (irAEs) [24]. In two melanoma trials, neoadjuvant anti-PDL1 and anti-CLTA4 combination treatments prior to, and continued after surgery resulted in grade ≥ 3 irAEs in 90 and 73% of patients [25, 26]. To reduce toxicity, subsequent trials in melanoma have successfully explored alternative strategies in the neoadjuvant setting, by lowering the ipilimumab dose to 1 mg/kg, or using a less frequent dosing schedule (NCT02977052, NCT03068455, NCT03241186, Checkmate -9LA trial). A pilot study in patients with NSCLC who received two doses of intravenous 3 mg/kg nivolumab prior to surgery, reported acceptable toxicity, with only one grade ≥ 3 event [27].

Based on findings of the abovementioned studies, we postulate that the addition of a short course of immunotherapy to CRT can improve both local and systemic disease control in locally advanced NSCLC, and increase the likelihood of achieving a pCR with acceptable toxicity. To investigate this hypothesis, we will evaluate whether the addition of ipilimumab-nivolumab to standard induction CRT for patients with resectable or borderline resectable stage IIB-III tumors is feasible, safe, and will result in increased rates of pCR and major pathologic response (MPR).

## Methods/design

### Trial design

This is a single center, single arm prospective phase II study, in which patients with pathology proven cT3-4 N0–1 M0 NSCLC will be eligible, except for those presenting with separate nodules in the same lobe as the primary tumor (T3) or ipsilateral lung (T4). Routine staging and diagnosis will be performed in accordance with current ESMO guidelines, including a tumor biopsy, pulmonary function tests, a whole body 18F-Fluoro-deoxy-glucose positron emission tomography/computed tomography (^18^F-FDG PET/CT) scan +/− esophageal ultrasound (EUS)/, endobronchial ultrasound (EBUS)/videomediastinoscopy and magnetic resonance imaging (MRI) of the brain, prior to start of treatment [8]. Patients will be discussed in a multidisciplinary tumor board (MDT), and eligible patients will be approached for study inclusion. CRT will be performed in accordance with the guidelines of ESMO and the EORTC [8, 28]. Patients will receive 1 cycle of ipilimumab and nivolumab (IPI/NIVO) on day 1 when radiotherapy commences, and 1 cycle of nivolumab will subsequently be administered 3 weeks after starting radiotherapy. After completion of CRT, a whole body ^18^F-FDG PET/CT scan will be repeated for restaging and exclusion of any new lesions, preferably 3 weeks after the last day of radiotherapy. Patients will undergo surgery approximately 6 weeks after finishing CRT.

### Objectives of the study

The primary objective is to assess the (1) safety of adding IPI/NIVO to CRT induction, and (2) the impact on pathological tumor response.

The secondary objectives are to assess the impact of adding IPI/NIVO to CRT on disease free and overall survival.

Exploratory objectives are the assessment of IPI/NIVO and CRT on tumor inflammation and the immune competence of tumor-draining lymph nodes (TDLN), and to explore the effects of IPI/NIVO and CRT and surgery on peripheral blood immune subsets distribution and phenotype. Finally, the possible impact of PD-1/PD-L1 status on the pathological tumor response, local and distant recurrence and overall survival will be evaluated.

### Endpoints of the study

Primary endpoints: (1) Safety will be assessed throughout the study. Any immune related adverse events (irAEs) will be registered including hyperthyroidism, hypothyroidism, adrenal insufficiency, hypophysitis, skin reactions, myositis, nephritis, pyrexia, pancreatitis, diabetes, increased transaminases, colitis, diarrhea, nausea, pleural effusion, dyspnea, pneumonia, or pneumonitis. Other adverse events deemed related to the study medication will also be registered. The causal association will be assessed. The severity of adverse events will be graded using the National Cancer Institute Common Terminology Criteria for Adverse Events (CTCAE). Also, morbidity after surgery and complications that lead to delay or canceling of CRT or surgery will be recorded. (2) Pathologic complete response (pCR) is defined as the absence of any viable tumor cells (ypT0N0M0) in the surgical resection specimen. MPR is defined as 10% or less viable tumor cells in the surgical resection specimen, and pathological response will be graded according to the Junker criteria and assessed in all resection specimens [27, 29].

Secondary endpoints: (1) Time to local or distant recurrence and (2) overall survival (OS) at 1 and 2 years.

### Sample size calculation

The primary outcome of pCR is defined as the absence of vital tumor in the resection specimen, e.g. the primary tumor site and the resected lymph nodes (hilar/mediastinal). The pCR after induction CRT is estimated to be 30%, based upon more than 100 institutional historical controls [11, 12]. Efficacy of adding IPI/NIVO to CRT will be assessed by testing whether the pCR at induction differs by 30% using a Z-test for a single proportion using a two-sided significance level of 5%. With a total number of 26 evaluable patients, the study will have 90% power to reject the null hypothesis in case pCR after IPI/NIVO plus induction CRT is 60%. To account for an expected drop-out rate of approximately 10%, we aim to include 29 patients.

### Patient selection

Patients with biopsy proven, clinically-staged T3-4N0–1 NSCLC, who are fit to undergo standard treatment in accordance with the INCREASE-protocol, will be screened for study eligibility. All patients will be discussed at the MDT, where tumor resectability will be assessed by at least two thoracic surgeons. Key inclusion and exclusion criteria are listed in Table 1.

**Table 1:** Inclusion and exclusion criteria for participation in the INCREASE trial

Inclusion criteria	Exclusion criteria
1. Histologically confirmed NSCLC 2. T3-4N0–1 tumors based on size or invasion into the thoracic wall, mediastinum, vertebra or diaphragm 3. Patients who are irresectable upfront, but expected to be resectable after chemoradiotherapy induction, as per multidisciplinary tumor board evaluation 4. Willing and able to provide written informed consent for the trial. 5. Aged above 18 years on day of signing informed consent. 6. Have measurable disease based on RECIST 1.1.^a^ 7. Have a performance status of 0–1 on the ECOG Performance Scale. 8. Demonstrate adequate organ function.	1. Known oncogenic drivers such as activating EGFR or BRAF mutations or ALK or ROS1 gene rearrangements 2. Prior surgery and/or radiotherapy on the ipsilateral thorax 3. Patients deemed inoperable 4. Active autoimmune disease. 5. Chronic systemic steroid therapy 6. Additional malignancy that is progressing or requires active treatment. Exceptions include basal cell carcinoma of the skin, squamous cell carcinoma of the skin, or in situ cervical cancer that has undergone potentially curative therapy. 7. Evidence of interstitial lung disease or active, non-infectious pneumonitis. 8. Active infection requiring systemic therapy. 9. A history of Human Immunodeficiency Virus (HIV) (HIV 1/2 antibodies). 10. Active Hepatitis B or C. 11. Psychiatric or substance abuse disorders that would interfere with cooperation with the requirements of the trial. 12. Has received prior therapy with an anti-PD-1, anti-PD-L1, anti-PD-L2, anti-CTLA-4 antibody, or any other antibody or drug specifically targeting T-cell co-stimulation or immune checkpoint pathways. 13. Patient is pregnant or breastfeeding, or expecting to conceive within the projected duration of the trial, starting with the pre-screening or screening visit through 23 weeks after the last dose of trial treatment.

^a^ RECIST: Eisenhauer EA, Therasse P, Bogaerts J, et al. New response evaluation criteria in solid tumours: revised RECIST guideline (version 1·1) Eur J Cancer. 2009;45:228–47

### Timeline

**Fig. 1:**
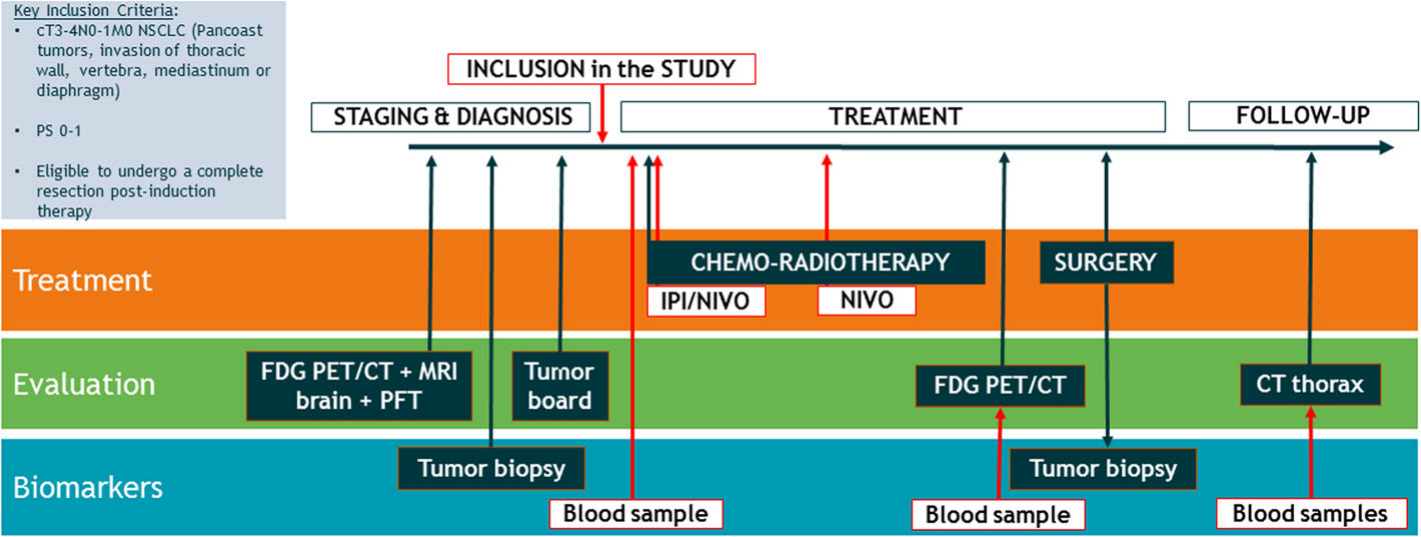
Key inclusion criteria, timeline and summary of the INCREASE protocol

Patients will be included in the trial, after obtaining written informed consent (Fig. 1). The protocol treatment commences with the first cycle of chemotherapy, concurrently with radiotherapy. Two cycles of chemotherapy will be given using either carboplatin with pemetrexed or carboplatin with etoposide for non-squamous and squamous histology, respectively. The recommended radiation dose will be 50 Gy, applied concurrently in 2Gy fractions, for 5–6 weeks. IPI/NIVO will be administered on day 1 of radiotherapy, and nivolumab alone will be administered for a second cycle after 3 weeks of radiotherapy.

### Procedures

#### Staging

An initial contrast-enhanced CT scan of the thorax, upper abdomen, an ^18^F-FDG PET/CT scan and magnetic resonance imaging (MRI) of the brain are mandatory for adequate staging. PET scans will be performed on EARL-accredited machines, being equipped with time-of flight (TOF) abilities, according to European Association of Nuclear Medicine (EANM) procedure guidelines for tumor imaging [30]. Enlarged lymph nodes (> 1 cm on CT) or ^18^F-FDG avid mediastinal and hilar lymph nodes will be examined by EUS/EBUS, or videomediastinoscopy, according to current guidelines on mediastinal staging of NSCLC [31]. A CT-guided transthoracic biopsy or trans-bronchial cytology/biopsy will be performed for histological confirmation of NSCLC and evaluation of PD-1/PD-L1 receptor status. Patients will be staged according the 8th edition of the TNM Classification of Malignant Tumours from the Union for International Cancer Control (UICC) [32].

#### Neoadjuvant therapy

##### Chemotherapy

Patients will be treated with CRT and surgery according to international guidelines [3]. Chemotherapy using platinum-doublets will be given in 3-weekly cycles. The choice of the chemotherapy agents will depend on tumor histology: non-squamous tumors will be treated with carboplatin ([Glomerular Filtration Rate (ml/min) + 25] x [5 mg/ml x min] IV) on day 1 of each cycle and pemetrexed (500 mg/m2 IV) on day 1 of each cycle. Dexamethasone (8 mg IV) on day 1 and 8 mg orally on days 0 and 2 of each cycle will be applied, folate (500 μg 1x/day orally) and hydroxocobalamin (1000 μg 1x/9 weeks intramuscularly) will be given during the whole course of the CRT.

Squamous tumors will be treated with carboplatin ([Glomerular Filtration Rate (ml/min) + 25] x [5 mg/ml x min] IV) on day 1 of each cycle and etoposide (100 mg/m2 IV on days 1, 2 and 3 of each cycle). Alternatively, etoposide may be given orally at 200 mg/m2 per day, on days 2 and 3 in selected patients. Dexamethasone (8 mg IV) on day 1 of each cycle.

A total of 2 cycles of a platinum-doublet will be given. However, in exceptional cases, a single cycle of chemotherapy alone can be started prior to the abovementioned concurrent IPI/NIVO/CRT treatment if the MDT decides that immediate initiation of systemic therapy is desirable for a patient, for example due to delays in completing RT planning.

##### Radiotherapy

Concurrent thoracic radiotherapy will be delivered in accordance with current guidelines with dose-fractionation schemes as described in the guidelines of the ESMO [8], using technical guidelines of the EORTC [28]. Intensity modulated radiotherapy (IMRT) will be delivered using once-daily fractions of 2 Gy for 5–6 weeks, resulting in the recommended dose of 50 Gy, and concurrent with the 1st or 2nd of 2 or 3 cycles of chemotherapy, respectively.

##### Immunotherapy

At 2 time points during CRT, patients will receive immunotherapy: At day 1 of the start of radiotherapy, patients will be treated with the combination of ipilimumab (1 mg/kg IV) and nivolumab (360 mg flat dose IV). The IPI/NIVO combination will be given concurrently with radiotherapy (± 1 week will be exceptionally acceptable if not otherwise feasible). At week 3 after start of RT, nivolumab (360 mg flat dose IV) will be administered for a second cycle.

#### Restaging

After completing CRT, all patients will undergo another ^18^F-FDG PET/CT scan according to our institutional protocol (preferably 3 weeks after completing radiotherapy), in order to exclude any new distant metastases that may have developed during CRT with IPI/NIVO treatment. The CT scan of the thorax will also be used for the response evaluation after induction therapy.

#### Surgery

Approximately 6 weeks after the last day of radiotherapy, patients will be operated at Amsterdam University Medical Center (AUMC), by thoracic surgeons experienced in complex pulmonary surgery, including post high-dose CRT [12, 33]. Surgery with curative intent will be performed under general anesthesia, with epidural pain relief, and will be performed by two surgeons. Anatomical resection (i.e. lobectomy or bilobectomy), with en-bloc resection of invaded structures e.g. thoracic wall, diaphragm or pericardium, will be performed together with systemic mediastinal nodal dissection. Routine clinical practice is intravenous antibiotics for 5 days after surgery (Ceftriaxone 2000 mg, once daily), routine bronchial stump coverage with well vascularized muscle flaps (e.g. intercostal muscle), early mobilization postoperatively, and aggressive dietary optimization (preferably starting before surgery).

### Follow-up

A routine control visit at the surgical outpatient clinic is planned approximately 2–3 weeks after surgery. Subsequently, routine outpatient follow-up visits will be scheduled every 3 months for the first 2 years after surgery, with pre-visit CT of the chest and upper-abdomen (including adrenals) every 6 months. Disease progression or recurrence suspected on radiological or clinical bases will be further investigated with additional imaging and tissue sampling where appropriate. Blood samples for translational research (see translational research section) will be collected at the first visit (e.g. before start of chemoradiotherapy), after induction therapy, i.e. 21 (±2) days after finishing CRT, and 3) in follow up at 12 (±1) weeks after surgery. For this purpose, 5 × 6 mL of blood will be drawn. Also, 3 additional blood samplings will be performed for isolation of plasma, isolation of peripheral blood mononuclear cells (PBMC’s), and storage for future biomarker analysis, i.e. at 6, 9, and 12 months post-surgery. A minimum of two-year follow-up is planned for all patients, with the study ending when the last patient completed follow-up.

### Adverse events

Adverse events (AEs) are defined as any new untoward medical occurrence or worsening of a preexisting medical condition in a clinical investigation participant to whom study drug was administered and that does not necessarily have a causal relationship with this treatment. An investigator who is a qualified physician will evaluate all adverse events according to Common Terminology Criteria for Adverse Events (CTCAE, National Cancer Institute, NCI). The causal relationship to the study drug will be assessed by a physician who will assess all AEs and annotate these AEs as related or not related to the study drug. All AEs will be recorded from the time the consent form is signed through 100 days following the last nivolumab application. A serious adverse event (SAE) is any untoward medical occurrence or effect that at any dose results in death or is life threatening (at the time of the event); requires new or prolongation of hospitalization, results in persistent or significant disability or incapacity, is a congenital anomaly or birth defect or any other important medical event that may jeopardize the subject or may require an intervention to prevent one of the outcomes listed above. Any component of a study endpoint that is considered related to study therapy should be reported as an SAE (e.g., overall survival is an endpoint in this study, and thus if death occurred due to anaphylaxis, anaphylaxis must be reported).

All AEs will be followed until they have abated, or until a stable situation has been reached. Depending on the event, follow up may require additional tests or medical procedures as indicated, and/or referral to the general physician or a medical specialist.

### Study discontinuation/withdrawal

Subjects may withdraw from the study at any time for any reason. The investigator can decide to withdraw a subject from the study for urgent medical reasons. Patients who withdraw from the study before surgery, and any patients that do not undergo surgery, will be replaced.

If treatment is discontinued for more than 6 weeks (see Appendix), the patient must be permanently discontinued from nivolumab therapy. The patient can still continue with the rest of the CRT and surgery treatment as planned. All AEs will be followed until they have abated, or until a stable situation has been reached.

The following rules were defined for definitely halting the study based on dose limiting toxicities (DLT). The DLT evaluation period is 6 weeks from the initiation of the combination of chemoradiotherapy and IPI/NIVO.

For immune related adverse events (irAE):

- The DLT based on irAE are defined as adverse events that are associated with exposure to IPI/NIVO and consistent with an immune adverse event:
  - ○ Grade ≥ 3 *pneumonitis*, that does not resolve in 1 week despite optimal supportive care
  - ○ Grade ≥ 3 IPI/NIVO-related *bronchospasm*, *allergic reaction, or infusion-related reaction* will be recorded and treated as per guidelines, however, will not count as DLT
  - ○ Grade ≥ 3 *diarrhea* that does not respond to the use of systemic steroids within 2 weeks
  - ○ Any Grade 3 non-skin irAE lasting > 1 week, except for *asymptomatic laboratory abnormalities*
- Based on these irAE, the study will stop:
  - ○ If in the first 6 patients, 3 or more patients develop a DLT based on irAE
  - ○ If in the first 9 patients, 4 or more patients develop a DLT based on irAE
  - ○ If in the first 15 patients, 6 or more patients develop a DLT based on irAE

For overall treatment related adverse events (trAE):

- The DLT based on treatment related adverse events (trAE) are defined as any grade ≥ 3 non-hematological toxicity and any grade 4 hematological toxicity that is probably, possibly or definitely related to the combination of chemoradiotherapy and IPI/NIVO.
  - ○ Grade 3 *fatigue* will not be classified as DLT, irrespective of duration
  - ○ Any grade 3 or higher non-hematologic *laboratory value* if:
    - ▪ the abnormality leads to hospitalization, or
    - ▪ life-threatening consequences and urgent intervention indicated
- Findings of a previous phase III trial in stage 3 NSCLC, standard of care (SoC) chemoradiotherapy using either platinum-pemetrexed or platinum-etoposide, reported grade 3–4 toxicity in 64 and 76.8% of patients, respectively [34]. We estimated the grade 3–4 trAE associated with SoC in the present study to be approximately 65% (a rather conservative guess) and that this could rise to 75%, by adding ipilimumab (1x) and nivolumab (2x), which would still be comparable to the platinum-etoposide arm of the PROCLAIM-study. Based on these data, the study will stop:
  - If in the first 8 patients, 7 or more patients develop a DLT based on trAE
  - If in the first 16 patients, 13 or more patients develop a DLT based on trAE

### Translational research: immune monitoring

The tumor microenvironment (TME) can be categorized according to the presence and distribution pattern of CD8+ T cells, as described by Chen and Mellman [35]. This T cell based approach classifies the TME into either immune desert (no T cells), immune excluded (T cells on the outer rims of the tumor fields), or inflamed (T cells infiltrating into the tumor fields) types. We hypothesize that IPI/NIVO combined with CRT will transform the TME into an inflamed (T cell infiltrated) type, coinciding with a decline in immune suppressive subsets such as Tregs, myeloid derived suppressor cells (MDSC), and M2-type macrophages, and in suppressive mediators such as IL-10, IDO, and arginase. Our study aims to explore this hypothesis and to characterize the changes in the TME, TDLN, and PBMC prior to, and after induction therapy and after surgery. Multiparameter flow cytometry and mass cytometry analyses of immune effector subset rates and activation state in peripheral blood before, during and after IPI/NIVO/CRT will be performed on a BD LSR Fortessa flow cytometer and a Helios mass cytometer. In addition, multi-parameter T cell fluorescence-activated cell sorting (FACS) analysis will be performed on dissociated tumor biopsies and lymph node samples, prior to treatment and a the time of surgery. The following subsets/populations will be included in these analyses provided sufficient cell yields are obtained (from the tumor and SLN samples): (memory/effector) CD4+ and CD8+ T cells, Tregs, granulocytic and monocytic MDSCs, conventional and plasmacytoid dendritic cells, and macrophages. All patients included in the study, will be asked to sign for participation in the translational research program, in which the following items will be investigated:

1. the presence and distribution pattern of tumor infiltrating lymphocytes (TIL’s) in the tumor microenvironment (TME) at baseline and after induction therapy.
2. correlation of post induction TIL’s with residual tumor cells/pCR.
3. changes in immune suppressive subsets/mechanisms in the TME and TDLN between pre and post induction.
4. correlation of these changes to residual viable tumor cells.

A baseline histological biopsy of the tumor lesion prior to start of induction therapy is mandatory, and the baseline biopsy and the resection specimen will be analyzed by routine hematoxylin and eosin (H&E) histology and multiplexed immunohistochemistry (IHC) staining panels for assessment of the immune infiltrate and viable tumor cells. Percentage of viable tumor cells will be scored. The resection specimen will be available from all patients, allowing comparison between the pre and post induction pathology.

### Data analysis

Descriptive statistics (proportions with 95% confidence intervals) will be used to summarize the primary endpoints of safety, pCR and MPR. Secondary endpoints of time to recurrence (local and distant recurrence), and overall survival (OS) will be summarized by means of Kaplan-Meier plots. The Z-test for a single proportion will be used for testing whether the proportion of patients with pCR after induction CRT differs from 30%. A two-sided significance level of 5% will be used.

## Discussion

For patients with a resectable T3 and T4 NSCLC, without mediastinal nodal involvement (N0–1), current guidelines recommend surgery +/− adjuvant chemotherapy, or induction chemoradiotherapy followed by resection [8]. This treatment approach results in overall 5-year survival rates of around 50%, and increases to 60–70% for patients with complete (or near complete) pathological response and radical resection [36, 37]. With pCR rates being less than 40%, there is room for improvements in induction therapy. The expected synergy between immunotherapy and CRT makes the addition of immunotherapy to neo-adjuvant protocols a promising approach. While several ongoing trials are exploring this neo-adjuvant immunotherapy, to the best of our knowledge no trial is investigating two types of neoadjuvant immunotherapy (PDL-1 and CTLA-4 blocking antibodies) in combination with standard concurrent chemoradiotherapy, in the treatment in locally advanced NSCLC.

Exploratory translational studies will assess the TME, TDLN and PBMC for immune subset distribution and activation. We hypothesize that IPI/NIVO combined with CRT will transform the TME into an inflamed type marked by an increase in tumor infiltrating lymphocytes (TIL’s).

There are few reports studying the optimal timing of surgery in neo-adjuvant protocols, and the duration of induction protocols used in published reports is not always clear. Most studies on neoadjuvant, multimodality studies including radiotherapy, report resections that were planned after approximately 4–8 weeks after the last dose of radiotherapy [11, 38]. This seems to be an optimal window, balancing radiation induced fibrosis and anti-tumor effects of radiotherapy, with the least risk of perioperative complications [39]. This strategy has been adapted in our institute since the introduction of trimodality for locally advanced NSCLC and sulcus superior tumors [12, 37].

The combination of chemotherapy, radiotherapy and immunotherapy is innovative, and we do not know the optimal timing of surgery yet. The ongoing LCMC3 study (NCT03927301) plans resection at approximately 3 weeks after the last of two cycles atezolizumab [40]. In a recent neoadjuvant NSCLC study, surgery was planned approximately 4 weeks after the first of two doses of nivolumab [27]. In the NADIM trial evaluating neoadjuvant nivolumab combined with carboplatin/paclitaxel, surgery is scheduled approximately 3–4 weeks after the last cycle of chemo/nivolumab induction [19].

As clear recommendations are lacking at present, we have set the time of surgery at 5–6 weeks after completing radiotherapy, although the optimal time-interval between neo-adjuvant immunotherapy (+/− CRT) and surgery is currently unknown and should be focus of future research.

To the best of our knowledge, INCREASE is the first study that will explore the safety of adding two types of immunotherapy to standard induction with CRT for patients with T3-4N0–1 NSCLC. Its effect on tumor control by expected increase of pCR and MPR rates, will be investigated by a translational research program.

## Data Availability

The datasets used and/or analysed during the current study are available from the corresponding author on reasonable request.

## Appendix

### IPI/NIVO treatment: criteria for dose delay and to resume treatment

The 2nd cycle of nivolumab should be delayed for the following:

- Any Grade ≥ 2 non-skin, immune related adverse event (irAE), with the following exceptions:
  - Grade 2 drug-related fatigue or laboratory abnormalities do not require a treatment delay
  - Any Grade 3 skin, drug-related AE
- Any Grade 3 drug-related laboratory abnormality, with the following exceptions for lymphopenia, leukopenia, AST, ALT, total bilirubin, or asymptomatic amylase or lipase:
  - Grade 3 lymphopenia or leukopenia does not require dose delay.
  - If a subject has a baseline AST, ALT, or total bilirubin that is within normal limits, delay dosing for drug-related Grade ≥ 2 toxicity.
  - If a subject has baseline AST, ALT, or total bilirubin within the Grade 1 toxicity range, delay dosing for drug-related Grade ≥ 3 toxicity.
  - Any Grade ≥ 3 drug-related amylase or lipase abnormality that is not associated with symptoms or clinical manifestations of pancreatitis does not require dose delay. The Investigator should be consulted for such Grade ≥ 3 amylase or lipase abnormalities
- Any AE, laboratory abnormality, or intercurrent illness which, in the judgment of the investigator, warrants delaying the dose of study medication. Subjects who require delay of nivolumab should be re-evaluated weekly or more frequently if clinically indicated and resume nivolumab dosing when re-treatment criteria are met.

### Subjects may resume treatment with nivolumab when the irAE resolve to Grade ≤ 1 or baseline value, with the following exceptions:

- Subjects may resume treatment in the presence of Grade 2 fatigue.
- Subjects who have not experienced a Grade 3 drug-related skin AE may resume treatment in the presence of Grade 2 skin toxicity.
- Subjects with baseline Grade 1 AST/ALT or total bilirubin who require dose delays for reasons other than a 2-grade shift in AST/ALT or total bilirubin may resume treatment in the presence of Grade 2 AST/ALT OR total bilirubin.
- Subjects with combined Grade 2 AST/ALT AND total bilirubin values meeting discontinuation parameters should have treatment permanently discontinued.
- Drug-related pulmonary toxicity, diarrhea, or colitis, must have resolved to baseline before IO treatment is resumed. Subjects with persistent Grade 1 pneumonitis after completion of a steroid taper over at least 1 month may be eligible for retreatment.
- Drug-related endocrinopathies adequately controlled with only physiologic hormone replacement may resume treatment.

## Abbreviations

18F-FDG PET: 18F-Fluoro-deoxy-glucose positron emission tomography
AEs: adverse events
AUMC: Amsterdam university medical center
CRT: chemoradiotherapy
CT: computed tomography
CTCAE: Common Terminology Criteria for Adverse Events
DLT: dose limiting toxicities
EARL: EANM (European Association of Nuclear Medicine) Research Ltd.
EBUS: endobronchial ultrasound
EORTC: European Organisation for Research and Treatment of Cancer
ESMO: European Society of Medical Oncology
EUS: esophageal ultrasound
FACS: fluorescence-activated cell sorting
Gy: Gray
H&E: hematoxylin and eosin
IHC: immunohistochemistry
IMRT: intensity modulated radiotherapy
IPI: ipilimumab
irAE: immune related adverse event
IV: intravenous
MDSC: myeloid derived suppressor cells
MDT: multidisciplinary tumor board
MPR: major pathological response
MRI: magnetic resonance imaging
NCI: National Cancer Institute
NIVO: nivolumab
NSCLC: non-small cell lung cancer
NTR: Netherlands Trial Registration
OS: overall survival
PBMC: peripheral blood mononuclear cell
pCR: pathological complete response
RT: radiotherapy
SAE: serious adverse event
SoC: standard of care
TDLN: tumor-draining lymph nodes
TIL: tumor infiltrating lymphocytes
TME: tumor microenvironment
TOF: time-of-flight
trAE: therapy related adverse events
